# Circulating (1 → 3)-β-D-Glucan as an immune activation marker decreased after ART in people living with HIV

**DOI:** 10.3389/fpubh.2022.981339

**Published:** 2022-09-14

**Authors:** Jingna Xun, Shuyan Guo, Yumin Xu, Rong Chen, Qi Tang, Xinyu Zhang, Danping Liu, Renfang Zhang, Yinzhong Shen, Li Liu, Jiangrong Wan, Jun Chen, Hongzhou Lu

**Affiliations:** ^1^Department of Infection and Immunity, Shanghai Public Health Clinical Center, Fudan University, Shanghai, China; ^2^State Key Laboratory of Genetic Engineering and Engineering Research Center of Gene Technology, School of Life Sciences, Fudan University, Shanghai, China; ^3^Shanghai Foreign Language School, Shanghai International Studies University, Shanghai, China; ^4^Department of Infectious Diseases, Ruijin Hospital, School of Medicine, Shanghai Jiao Tong University, Shanghai, China; ^5^Department of Infectious Diseases and Nursing research institution, National Clinical Research Center for Infectious Diseases, The Third People's Hospital of Shenzhen, Guangdong, China

**Keywords:** HIV, (1 → 3)-β-D-Glucan, immune activation, microbial translocation, ART

## Abstract

**Background:**

Plasma level of polysaccharide (1 → 3)-β-D-Glucan (βDG), as a diagnostic marker of invasive fungal infection has been reported to be elevated in people living with HIV (PLWH). We assessed the association of circulating βDG to inflammation and systemic immune activation and the effect of antiretroviral therapy (ART) on βDG in PLWH.

**Method:**

Plasma and peripheral blood monocular cell samples from 120 PLWH naive to ART and after 1 year's ART were collected. Plasma levels of βDG, markers of bacterial translocation, gut damage, and cellular immune activation were quantified.

**Result:**

The plasma βDG levels were negatively correlated with CD4+ T cells count (*r* = −0.25, *p* = 0.005) and positively with HIV viral load (*r* = 0.28, *p* = 0.002) before ART. It was also positively correlated with immune activation markers, including PD-1 expression on CD4+ T cell (*r* = 0.40, *p* = 0.01) and CD8+ T cell (*r* = 0.47, *p* = 0.002), as well as HLADR+CD38+ co-expression on CD8+ T cell (*r* = 0.56, *p* = 0.0002), but not with the plasma levels of LPS (*r* = 0.02, *p* = 0.84), LPS binding protein (LBP, *r* = 0.11, *p* = 0.36), soluble LPS receptor sCD14 (*r* = 0.04, *p* = 0.68), intestinal fatty acid binding protein (IFABP, *r* = −0.12, *p* = 0.18), and regenerating islet-derived protein 3α (REG3α, *r* = 0.18, *p* = 0.06). After 1 year's ART, the levels of βDG were significantly decreased compared to that in pre-ART (1.31 ± 0.24 Log10 pg/ml *vs*. 1.39 ± 0.18 Log10 pg/ml, *p* < 0.001).

**Conclusion:**

The level of plasma βDG was associated with cellular immune activation and decreased after ART in PLWH, suggesting it could serve as a biomarker of immune activation and efficacy monitoring.

## Introduction

Increased T-cell turnover, elevated serum levels of pro-inflammatory cytokines and chemokines, and altered gut microbiome translocation were major characteristics of HIV infection (1–3). Microbial translocation which occurs partially due to the increased intestinal permeability leads to systemic immune activation in chronic HIV infection (4, 5). While most previous studies reported that levels of markers of bacterial translocation, mostly lipopolysaccharide (LPS) were elevated, recent studies also suggest that fungus may also translocate from gut to blood in people living with HIV (PLWH) (6, 7).

Fungal cell walls contain polysaccharides that are absent in humans. As one of the major components of fungal cell walls, (1 → 3)-β-D-Glucan (βDG), is a useful target for assessing invasive fungal in circulation (8). Currently, the utility of βDG assays represents a promising tool for the diagnosis of invasive fungi such as Candida albicans, Aspergillus fumigatus, H. capsulatum, and T. marneffei are common in people with HIV (PLWH) (9). Several studies indicate that βDG can initiate immune recognition, induce the production of pro-inflammatory cytokines and chemokines, and trigger the immunity pathway (10, 11).

The levels of immune activation aim at identifying associations between the relevant biomarkers and clinical outcomes. In our report, we accessed the association of βDG with other immune activation markers, bacterial translocation, and gut damage. We further quantified the dynamic changes of levels of βDG in PWLH initiating ART.

## Methods

### Study and design population

Blood samples from participants in a prospective, randomized, clinical trial were collected (12). All the participants' PLWH were diagnosed by measuring plasma HIV-1 antibody and confirmed by the Western blot method of the Chinese Center for Disease Control and Prevention, aged from 18 to 60 years old. Our study excluded those who had obvious abnormalities after physical imaging examinations, a clear medical history of the central nervous system, cardiovascular system, digestive system, respiratory system, genitourinary system, and blood system, and who were diagnosed with opportunistic infections and tumors. All the participants were enrolled before ART, then received TDF/3TC/EFV regimen treatment and followed up during the first year's ART. This study was approved by the SPHCC Ethics Committee (2016-S054-01). Informed consent was obtained from all the participants.

### Measurement of plasma βDG level, bacterial translocation, gut damage markers, and soluble inflammatory markers

Sequential blood samples from a total of 120 participants were analyzed. Plasma βDG was measured by the Fungitell Limulus Amebocyte Lysate (LAL) assay (Associates of Cape Cod, Inc, East Falmouth, MA, USA) according to the manufacturer's instruction. Enzyme-linked immunosorbent assays (ELISAs) were performed to quantify plasma LPS (CUSABIO, Wuhan, Hubei, China), LPS binding protein (LBP, Hycultbiotech, Uden, Netherlands), soluble LPS receptor CD14 (sCD14), intestinal fatty acid binding protein (IFABP), regenerating islet-derived protein 3α (REG3α), and soluble CD163 (R&D Systems, Minneapolis, MN, USA).

### Surrogate markers of immune activation determined by flow cytometry

To detect the correlation between βDG levels and immune activation before ART, we investigated T-cell activation by measuring PD-1 expression on CD4+ and CD8+ T cells (13–15) and also detected the co-expression of CD38+ and HLA-DR+ on CD8+ T cells. Blood samples from 39 out of the 120 participants were used to determine the level of immune activation. Frozen peripheral blood mononuclear cells were rapidly thawed and stained with the following antibodies: CD3 APC-H7, CD4 FITC, CD8 APC, CD38 PE-Cy7, HLA-DR PerCP-Cy5.5, PD-1 PE, and live/dead FVS510 (all from BD Biosciences, San Jose, CA, USA) for 20 min at 4°C. Cells were fixed in 1% paraformaldehyde and analyzed within 24 h of staining. Data were analyzed using FlowJo software version 10 (FlowJo, LLC, Ashland, Oregon).

### Statistics analyses

Data were analyzed using IBM SPSS version 23 and GraphPad Prism 8.0 software. Continuous data with normal distribution were expressed as means ± standard deviation (χ ± *SD*) and compared using *t*-tests; continuous data with skewed distribution were expressed as median (inter-quartile range, IQR) and compared using the Kruskal–Wallis test. Categorical data were expressed as frequencies and percentages and compared using the chi-square (χ^2^) test. The correlation analysis was performed using the non-parametric Spearman test. The *p* < 0.05 was statistically significant.

## Results

### Study participant characteristics

Among all the participants, the median (IQR) age of participants was 28.5 (25–35) years and 92.5% were male. The median Pre-ART HIV RNA was 4.47 (4.02–4.81) Log10 copies/ml. The median CD4 T-cell count was 287.00 (193.00–411.00) cells/μl, and it was improved after ART to 470.50 (338.00–657.75). All the participants reached viral suppression after 1 year's ART. All the characteristics of the 120 participants were described in Table 1.

**Table 1:** Clinical characteristics of study population.

**Characteristics**	***N*** ^*^= 120
Age at ART initiation, y, median (IQR)	28.50 (25.00–35.00)
Male sex, No. (%)	92.5
Standard dose. No. (%)	50.83
Pre-ART CD4 T-cell count, cells/μL, median (IQR)^a^	287.00 (193.00–411.00)
Pre-ART CD4/CD8 ratio, median (IQR)^b^	0.29 (0.19–0.40)
Pre-ART HIV RNA, log10 copies/mL, median (IQR)	4.47 (4.02–4.81)
On-ART CD4 T-cell count, cells/μL, median (IQR)^c^	470.50 (338.00–657.75)
On-ART CD4/CD8 ratio, median (IQR)^c^	0.61 (0.40–0.85)

* The number of participants could be changed based on different characteristics.

a n = 119;

b n =118;

c n = 117.

ART, antiretroviral therapy; HIV, human immunodeficiency virus; IQR, interquartile range; VL, viral load.

### βDG levels were correlated with markers of immune activation, but not with markers of bacterial translocation and gut damage

**Figure 1:**
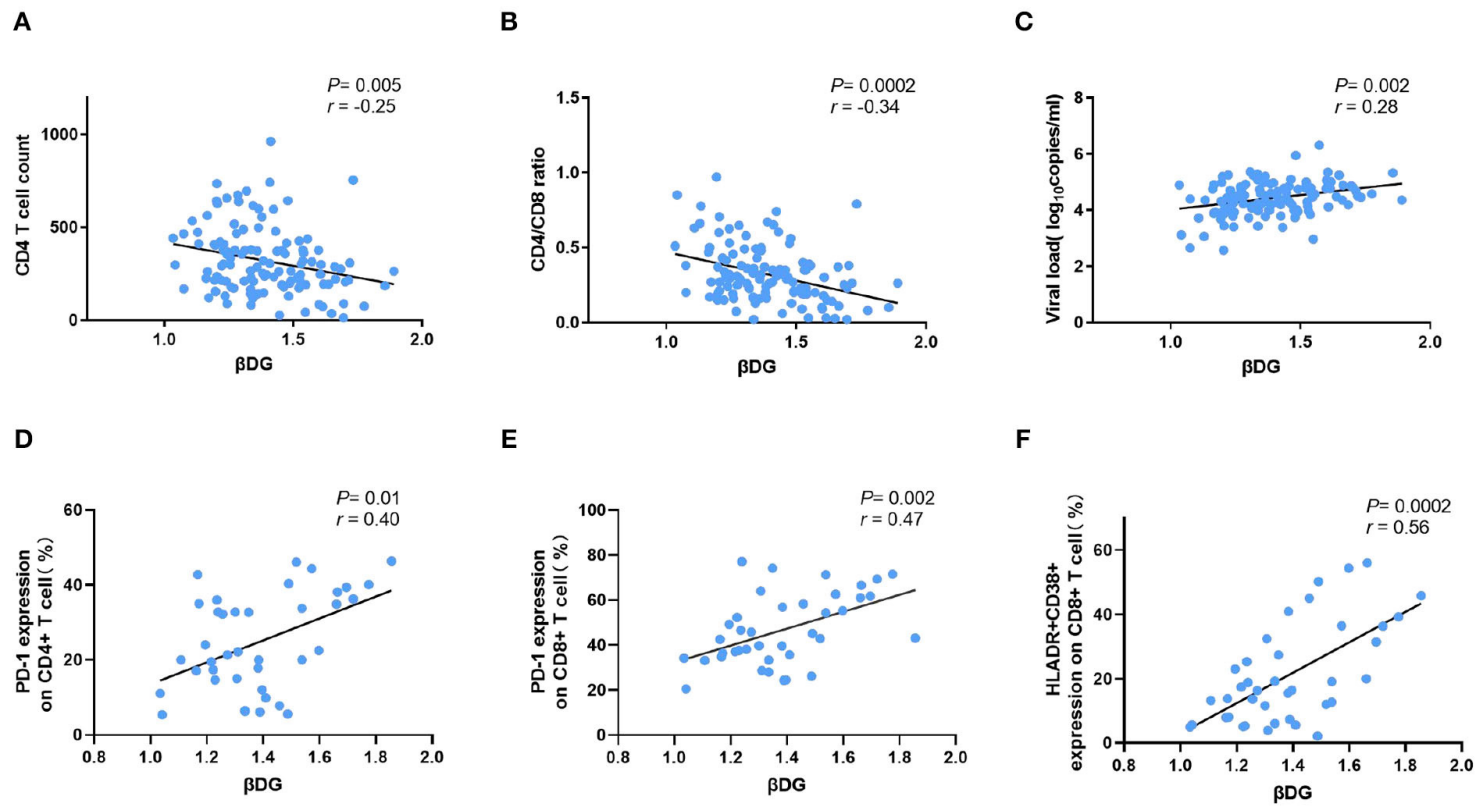
Comparison of βDG with markers of CD4 T cells, viral load, and immune activation markers. **(A)** β-D-glucan (βDG) levels were correlated with CD4 T cell count (*n* = 119). **(B)** βDG levels were correlated with CD4/CD8 ratio in untreated patients (*n* = 118). **(C)** βDG levels were correlated with plasma viral load at baseline (*n* = 120). **(D)** There were positively correlations among BDG levels and the expression of PD-1 on CD4+ T cell (*n* = 39), **(E)** the expression of PD-1 on CD8+ T cell (*n* = 39), **(F)** the co-expression of HLADR and CD38 on CD8+ T cell (*n* = 39). All the analysis using by nonparametric spearman test.

**Figure 2:**
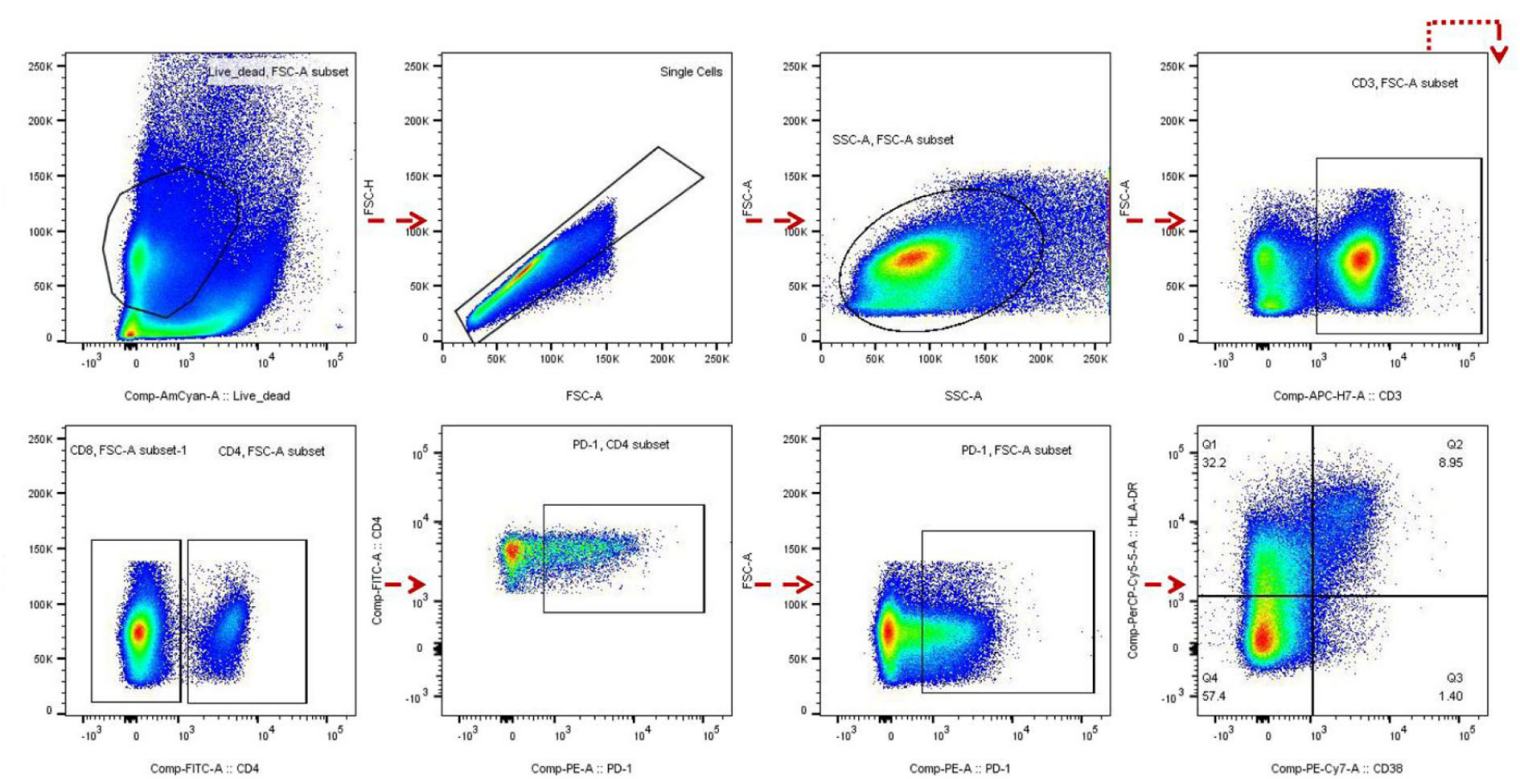
Flow cytometry analysis. The gating strategy of PD-1 and HLADR+ CD38+ expression in CD4 and/or CD8 cells of HIV infected patients before and after antiretroviral therapy (ART) was analyzed by flow cytometry.

In PLWH naive to ART, the βDG levels were negatively correlated with CD4 T cell count (*r* = −0.25, *p* = 0.005; Figure 1A) and pre-ART CD4/CD8 ratio (*r* = −0.34, *p* = 0.0002; Figure 1B). Conversely, the baseline plasma viral load (*r* = 0.28, *p* = 0.002; Figure 1C), the frequency of PD-1 expressing on CD4+ and CD8+ T cells (*r* = 0.40, *p* = 0.01; *r* = 0.47, *p* = 0.002; Figures 1D,E), the co-expression of CD38 and HLA-DR on CD8+ T cells (*r* = 0.56, *p* = 0.0002; Figure 1F), were all positively correlated with level of βDG. The gating strategy of flow cytometry utilized to identify and characterize the various immune populations is shown in Figure 2.

We then explored the association between plasma levels of βDG with markers of bacterial translocations. Interestingly, plasma level of βDG was not correlated with any of markers of bacterial translocations, such as plasma levels of LPS (*r* = 0.02, *p* = 0.84), LBP (*r* = 0.11, *p* = 0.36), sCD14 (*r* = 0.04, *p* = 0.68), nor did associated with markers of gut damage, such as IFABP (*r* = −0.12, *p* = 0.18) and REG3α (*r* = 0.18, *p* = 0.06) (Table 2).

**Table 2:** Association of microbial translocation and gut damage markers with β-D-glucan (βDG) levels pre- antiretroviral therapy (ART).

**Biomarkers**	**Pre-ART**		
	**Results**	**Spearman correlation with βDG**	***p*** **-value**
βDG (log10 pg/ml)	1.39 ± 0.18	–	–
LBP (log10 ng/ml)	3.74 ± 0.27	0.11	0.36
IFABP (log10 pg/ml)	3.11 (2.87–3.32)	−0.12	0.18
LPS (log10 pg/ml)	1.49 (1.33–1.67)	0.02	0.84
sCD14 (log10 pg/ml)	6.39 (6.00–6.66)	0.04	0.68
Reg3α (log10 pg/ml)	4.14 (3.90–4.42)	0.18	0.06

### Levels of βDG and markers of immune activation, but not markers of bacterial translocation were decreased after ART

**Figure 3:**
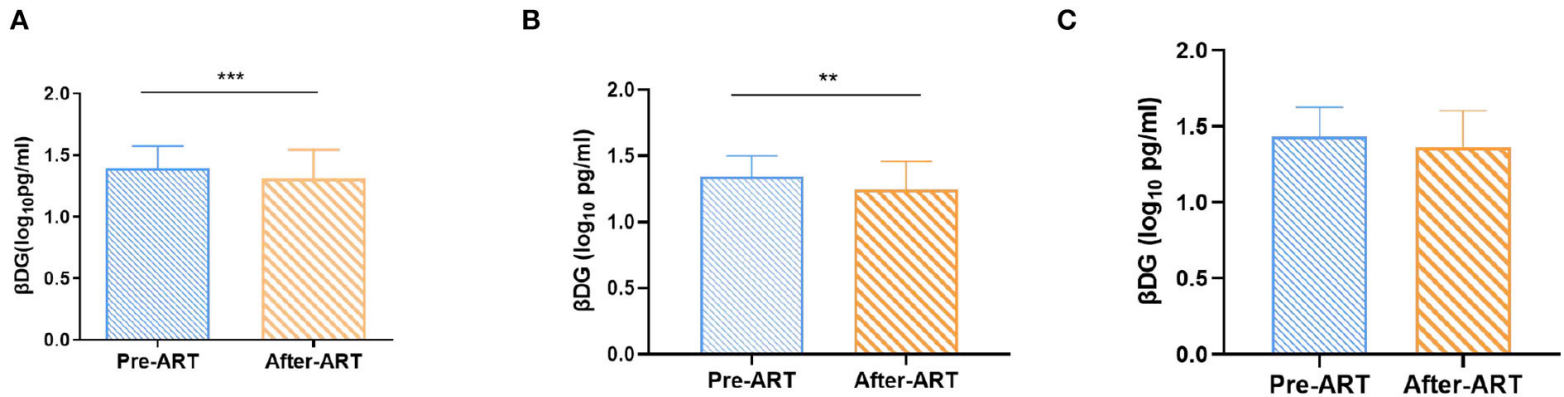
Changes of βDG levels previous and after ART. **(A)** Total change of βDG level after ART (*n* = 120). **(B)** Longitudinal analysis showed a decreased in βDG levels after ART in the CD4+ T cell count > 300 cells/ul group (*n* = 65). **(C)** Longitudinal analysis showed that after ART, there was no change of βDG levels in the CD4+ T cell count ≤ 300 cells/ul group (*n* = 55). All the analysis using by paired *t* test. ***p < 0.001, **p < 0.01.

After ART, the βDG levels were significantly decreased compared to pre-ART (1.31 ± 0.24 Log10 pg/ml vs. 1.39 ± 0.18 Log10 pg/ml, *p* < 0.001; Figure 3A). Considering that the median (IQR) of CD4+ T cell count was 287.00 (193.00–411.00) cells/μl, and the βDG levels were negatively correlated with CD4+ T cell count at baseline (*r* = −0.25, *p* = 0.005); participants were then further classified into two subgroups depending on the CD4+ T cell count (300 cells/μl). We evaluated the correlations between βDG levels and ART in CD4 > 300 cells/μl group and CD4 ≤ 300 cells/μl group, respectively. The βDG levels decreased notably after ART in CD4 > 300 cells/μl group (1.34 ± 0.16 Log10 pg/ml vs. 1.24 ± 0.22 Log10 pg/ml, *p* = 0.003; Figure 3B) but no obvious change in CD4 ≤ 300 cells/μl group (1.43 ± 0.19 Log10 pg/ml vs. 1.36 ± 0.24 Log10 pg/ml, *p* = 0.06; Figure 3C).

**Figure 4:**
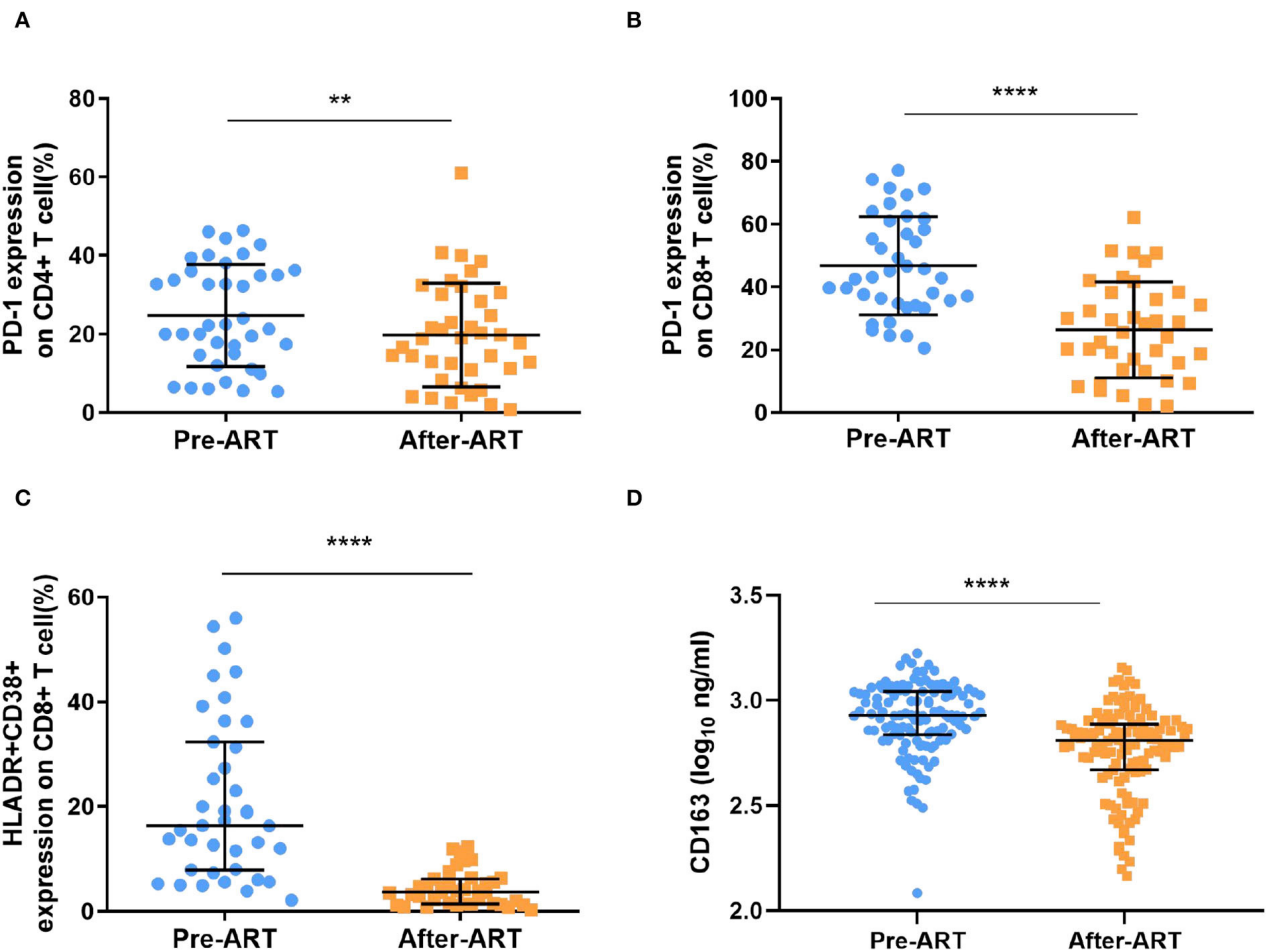
Markers of immune activation after ART. **(A,B)** The percentage of PD-1 expressing on CD4+ T cells and CD8+ T cells were significantly decreased after ART (*n* = 39, paired Kruskal–Wallis test; paired *t* test). **(C)** the expression of HLADR+ and CD38+ within CD8+ T cells was significantly reduced (*n* = 39, paired Kruskal–Wallis test). **(D)** The levels of plasma CD163 was detected by ELISA, which decreased significantly (*n* = 120, paired Kruskal–Wallis test). ****p < 0.0001, **p < 0.01.

Next, we explored the changes in immune activation markers after ART. The levels of PD-1 percentage among CD4+ T cells have a slighter reduction [22.20 (14.70–36.00) vs. 18.80 (10.90–30.10), *p* = 0.007, Figure 4A], while the percentage of PD-1 expression on CD8+ T cell have more significant effect during ART [43.10 (34.80–61.00) vs. 25.60 (13.70–38.30), *p* < 0.0001, Figure 4B]. The frequency of activated CD8+ T cells, as measured by co-expression of CD38+ and HLADR+, was significantly reduced during ART [16.40 (7.93–32.40) vs. 3.67 (1.45–6.18), *p* < 0.0001, Figure 4C]. Additionally, as a marker of immune-activated disease progression, the plasma level of CD163 also decreased significantly after ART [2.93 (2.84–3.04) Log10 pg/ml vs. 2.81 (2.67–2.88) Log10 pg/ml, *p* < 0.0001, Figure 4D].

**Figure 5:**
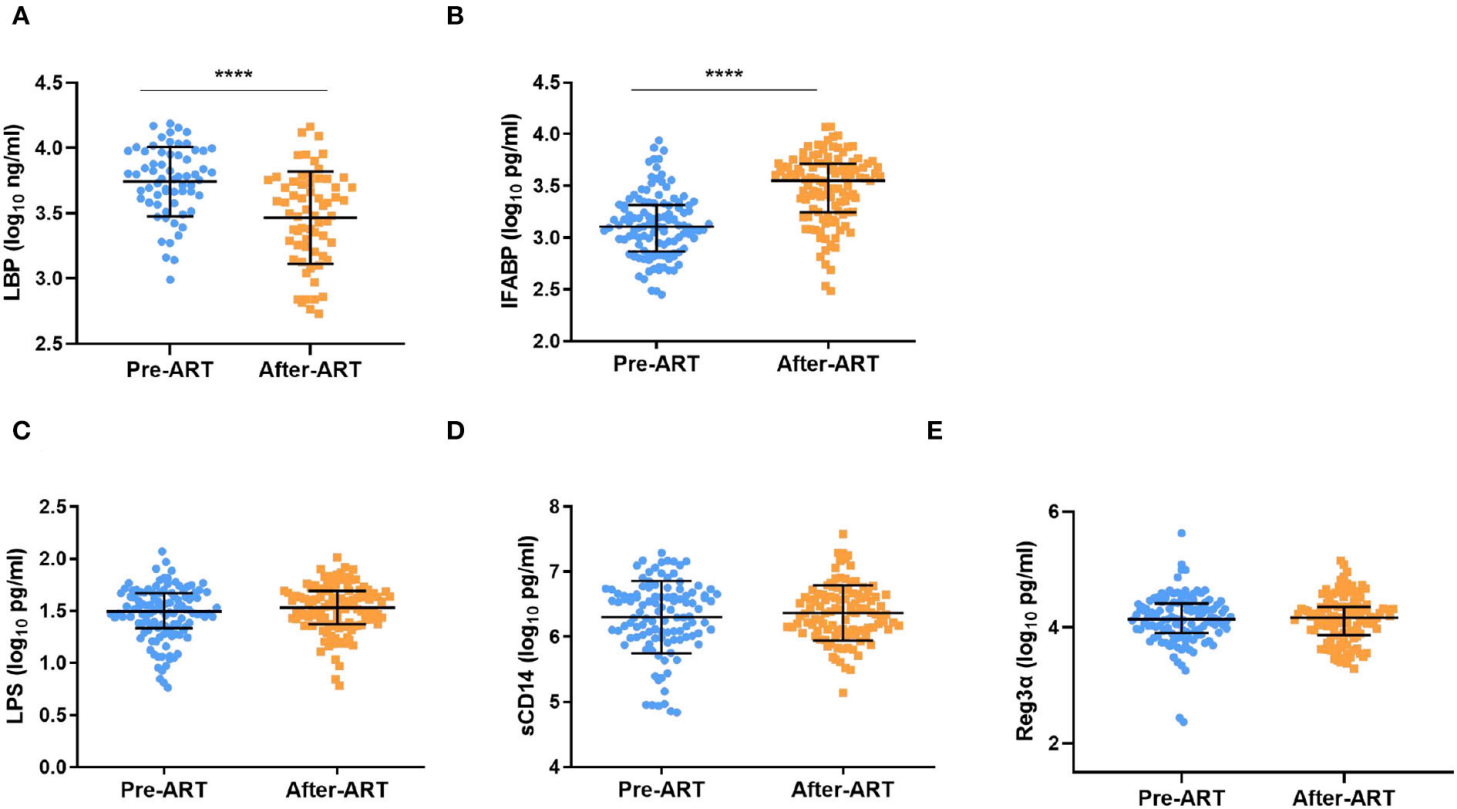
Serum levels of β-D-glucan (BDG), LBP, LPS, sCD14, and Reg3αin PLWH Pre-ART and after-ART. **(A)** βDG concentrations decreased slightly after ART (*n* = 65, paired *t* test). **(B)** The level of LBP decreased significantly after ART (*n* = 117, paired Kruskal–Wallis test);. **(C–E)** the plasma level of LPS (*n* = 119), sCD14 (*n* = 110), and Reg3α (*n* = 120) remain unchanged from pre ART to after ART (**C–E**: paired Kruskal–Wallis test). ****p < 0.0001.

For the bacterial translocation and gut damage markers, we only found that LBP decreased significantly after ART (3.74 ± 0.27 Log10 ng/ml vs. 3.47 ± 0.35 Log10 ng/ml, *p* < 0.0001, Figure 5A). Interestingly, the IFABP level increased after ART [3.11 (2.87–3.32) Log10 pg/ml vs. 3.55 (2.49–3.72) Log10 pg/ml, *p* < 0.0001, Figure 5B]. There was no significant change among the level of LPS, sCD14, and Reg3α after ART [(1.49 (1.33–1.67) Log10 pg/ml vs. 1.53 (1.37–1.70) Log10 pg/ml, *p* = 0.07; 6.39 (6.00–6.66) Log10 pg/ml vs. 6.36 (6.09–6.65) Log10 pg/ml, *p* = 0.28; 4.14 (3.90–4.42) Log10 pg/ml vs. 4.17 (3.87–4.36) Log10 pg/ml, *p* = 0.28, Figures 5C–E].

## Discussion

In this study, we found that the βDG levels were positively correlated with markers of immune activation, which suggested that βDG can be used as a marker of immune activation in PWLH. We also demonstrated that the level of βDG decreased after ART, especially in PLWH with CD4+ T cell count >300 cells/μl.

We showed that plasma βDG level was significantly associated with other known immune activation markers. This is consistent with previous studies (6, 16, 17). Morris et al. (6) proved that the increased plasma βDG in PLWH patients was related to the levels of IL-8, TNF–α, and the frequency of CD38 + and HLA-DR + CD8 + T cells; Hoenigl et al. (16) study showed that the level of plasma βDG was positively correlated with the level of IL-6, another immune activation marker. Importantly, Ramendra et al. (17) showed that the level of βDG was correlated with the expression of Dectin-1 and NKp30 in PLWH, and the Dectin-1 and NKp30 were associated with the activation of monocytes and NK cells *in vitro*. In our study, plasma βDG levels were positively correlated with the frequency of PD-1 expression on activated CD4 or CD8 T cells, and HLADR+CD38+ co-expression on CD8 T cells, illustrating that the βDG can directly or indirectly result in polyclonal T-cell activation (4, 18). All of these results, both *in vitro* and *in vivo* suggest that βDG could induce immune activation in PLWH and may severe as a new marker to predict HIV disease progression. Although long-term ART can control the HIV viral load at a very low level, the development of non-AIDS events in the body is also a great challenge for PLWH. Judging whether βDG is related to innate immune activation, inflammation, and the increased risk of non-AIDS events in ART in basic research and clinical trials will help to understand the new treatment strategies of βDG for AIDS and non-AIDS events.

All LPS, LBP, and sCD14 have been used to indicate bacterial translocation (19, 20), REG3α as a marker of gut damage (19, 21), while IFABP was used to characterize intestinal cell death (22). Surprisingly, there were no correlations between βDG and markers of bacterial translocation and gut damage in our study, indicating that βDG-induced immune activation may be independent of bacterial translocation and could be non-parallel with bacterial translocation. However, several published studies showed that the level of βDG in PLWH was associated with bacterial translocation markers including LPS, LBP, sCD14, and I-FABP (7, 23–25). These differences may be explained by different times of ART initiation and variability of bacterial translocation markers.

For the first time in a longitudinal study, we found that the level of βDG was decreased after ART. Few previous studies have investigated the effects of ART on βDG. In the study with 21 patients followed up after ART, Mehraj et al. (25) reported that βDG levels remained unchanged after 24 months of ART. However, in a randomized clinical trial, where PLWH received either Tenofovir-emtricitabine (TDF/FTC) plus atazanavir-ritonavir (ATV/r), darunavir-ritonavir (DRV/r), or raltegravir (RAL) over 96 weeks, Dirajlal-Fargo et al. (26) found that there was an overall increase in βDG over 96 weeks. These different results may be attributed to different times of ART initiation. In our study, the CD4+ T cells count was relatively low when compared to the above-mentioned two studies. Nevertheless, in our subgroup analysis, the level of βDG decreasing was only observed in PLWH with high CD4 T cell count. Interestingly, all the PLWH received protease inhibitors or integrase inhibitors-based ART in Dirajlal-Fargo's study, while all participants in our study received efavirenz-based ART. It is known that efavirenz has potential antimicrobial activity which could alter the gut microbiome (27); therefore, whether this different regimen could impact plasma βDG levels needs further investigation.

There were several limitations to our study. We neither collected nor controlled the diet habit of the participants as some food; especially mushrooms may impact levels of βDG (28). Second, PBMCs were only collected in part of the participants for flow cytometry analysis. Third, our participants are relatively young and mainly male, limiting our results to be generalizable to other PWLH.

## Conclusion

Our study provides evidence that plasma βDG level is a marker of immune activation which decreased after ART. Therefore, it may be useful to monitor HIV disease progression and therapeutic responses. Further studies are needed to further confirm this application.

## Data availability statement

The raw data supporting the conclusions of this article will be made available by the authors, without undue reservation.

## Ethics statement

The studies involving human participants were reviewed and approved by Shanghai Public Health Clinical Center Ethics Committee (2016-S054-01). The patients/participants provided their written informed consent to participate in this study.

## Author contributions

JC and HL were responsible for designing the study and revising the manuscript. JX, SG, and YX handled the specimens, did the experiments, analyzed the data, and wrote the manuscript. RC, RZ, YS, LL, and JW participated in the conduct of the study, including the recruitment, and follow-up of participants. RC was responsible for collecting the clinical data. QT directed and helped with the data analysis. XZ and DL assisted with ELISA experiments. All authors reviewed the article for intellectual content, contributed to the article, and approved the submitted version.

## Funding

This work was supported by Shanghai Shenkang Hospital Development Center (16CR1018A), the People's Republic of China (2017ZX09304027 and 2017ZX10202101), the Shanghai Science and Technology Committee (19YF1441300), and Shanghai Municipal Health Commission (2019-72; 20184Y0007).

## Conflict of interest

The authors declare that the research was conducted in the absence of any commercial or financial relationships that could be construed as a potential conflict of interest.

## Publisher's note

All claims expressed in this article are solely those of the authors and do not necessarily represent those of their affiliated organizations, or those of the publisher, the editors and the reviewers. Any product that may be evaluated in this article, or claim that may be made by its manufacturer, is not guaranteed or endorsed by the publisher.
